# CYFRA 21-1 is an early predictor of chemotherapeutic effectiveness in advanced nonsmall cell lung cancer: An observational study

**DOI:** 10.1097/MD.0000000000005748

**Published:** 2016-12-30

**Authors:** Tongwei Zhao, Ying Jin, Guangyun Mao, Yaping Wei, Guoqing Wu, Xiao Ye, Yonglie Zhou, Guorong Yuan, Liang Gao, Yupeng Hong, Yun Chen, Chaojin Hong, Hongying Zhou, Dan Su, Zhiquan Qin, Liqin Lu

**Affiliations:** a Department of Medical Oncology, Zhejiang Provincial People's Hospital; b Department of Medical Oncology, Zhejiang Cancer Hospital; c Department of Preventive Medicine, School of Environmental Science & Public Health, Wenzhou Medical University; d Center on Clinical & Epidemiological Eye Research, the Affiliated Eye Hospital of Wenzhou Medical University; e Department of Endocrinology, Zhejiang Provincial People's Hospital; f Clinical Laboratory Center, Zhejiang Provincial People's Hospital, Zhejiang, P.R. China.

**Keywords:** carcinoma, chemotherapy effectiveness, CYFRA21-1, nonsmall cell lung cancer, predictive factor

## Abstract

Serum cytokeratin 19 fragment (CYFRA21-1) has been found to be a useful prognostic marker in lung cancer. Previous studies have revealed that change in CYFRA21-1 synchronously predicted therapeutic effectiveness in advanced nonsmall cell lung cancer (NSCLC) after the second cycle of chemotherapy. The objective of this study was to investigate the early predictive value of percentage change in serum CYFRA21-1 from pretreatment to completion of the first cycle of chemotherapy for chemotherapeutic effectiveness in advanced NSCLC patients.

Ninety-seven advanced NSCLC patients with elevated serum CYFRA21-1 level (≥3.8 μg/L), who received 2 platinum-containing drugs, were included in this retrospective study. Serum CYFRA21-1 had been assayed before and after the first cycle of chemotherapy. To evaluate the effectiveness of chemotherapy, patients were allocated to disease control (DC) and progressive disease groups. The percentage changes of serum CYFRA21-1 concentration before and after first-cycle chemotherapy that occurred in each group were evaluated for their ability to predict achievement of radiologic DC, that is, to predict therapeutic effectiveness.

The percentage change of serum CYFRA21-1 and the prevalence of ≥5% weight loss were higher in patients with progressive disease than in those with DC. The differences in other clinical and pathological variables including age, sex, Eastern Cooperative Oncology Group (ECOG) performance status, cigarette smoking, histological type, gross type, clinical stage, and chemotherapy regimens of the 2 groups were not significant. Both multiple generalized linear model analysis and linear trend tests indicated that the percentage change of serum CYFRA21-1 concentration was independently and negatively linked to the effectiveness of chemotherapy for NSCLC (*P* < 0.01). The area under the receiver-operating characteristic curve of the percentage change in prediction of DC was 0.84 and the optimal cut-off value was17.5% (*P* < 0.001).

The percentage change of serum CYFRA21-1 after completing the first cycle of chemotherapy was predictive of treatment effects and might be helpful in making early decisions to change chemotherapy regimens in patients with advanced NSCLC.

## Abstract 2

Supplemental Digital Content is available in the text

## 1 Introduction

Lung cancer is the leading cause of cancer death in men worldwide,^[1]^ and in both men and women in China.^[2]^ Nonsmall cell lung cancer (NSCLC) accounts for 70% to 80% of the lung cancer diagnoses, and 70% of those are diagnosed when advanced stage is reached (stage IIIB/IV).^[3]^ Current clinical guidelines for first-line therapy and subsequent therapy of NSCLC from the National Comprehensive Cancer Network (NCCN)^[4]^ highlight the significant progress made since 1997 when there was only 1 option for first-line therapy.^[5]^ Chemotherapy is an important choice as the first-line therapy, especially for those whose test results of epidermal growth factor receptor (EGFR) mutation and anaplastic lymphoma kinase (ALK) gene rearrangement are negative.^[4]^

Patient response to treatment of advanced NSCLC is assessed radiologically as per the Response Evaluation Criteria in Solid Tumors (RECIST) Version 1.1^[6]^ after the second cycle of chemotherapy. Disease control (DC), classified as complete response (CR), partial response (PR), or stable disease (SD), is considered as a more reliable predictor of survival than objective response (OR) rate, and provides an early assessment of outcome.^[7]^ Oncologists usually continue first-line advanced NSCLC regimens for patients with DC and switch to subsequent therapy for those with progressive disease (PD) after 2 cycles of chemotherapy. This means that some patients with a PR receive less effective chemotherapy drugs after the second cycle of chemotherapy. Therefore, it would be helpful to determine as early as possible whether patients will get benefit from first-line chemotherapy. Early adjustment of the initial therapy would avoid unnecessary side effects and save time and cost. Currently, there are no tools that effectively predict the response to chemotherapy, especially DC after the first cycle of chemotherapy.

Tumor-associated serum markers have predictive and prognostic value in patients being treated for malignancies. Our previous studies demonstrated that serum dehydrogenase, C-reactive protein, and albumin had independent prognostic value in nasopharyngeal carcinoma and NSCLC.^[8–10]^ Cytokeratin 19 fragment (CYFRA21-1) is expressed in the cytoplasm of epithelial tumor cells, including NSCLC,^[11]^ and Vollmer et al^[12]^ found that serum CYFRA21-1 level was associated with tumor stage, patient prognosis, and surgical resection of tumors, and reflected tumor burden. Serum CYFRA21-1 level has also been shown to predict treatment effectiveness and prognosis in patients treated with surgery,^[13,14]^ chemotherapy,^[15–17]^ targeted therapy,^[18–20]^ and concurrent chemoradiation.^[21]^ Previous studies of the association of change in serum CYFRA21-1 with response to chemotherapy, which focused on synchronous or early prediction of OR, found that it did have predictive value.^[12–25]^ A few investigations have reported the predictive value of change in serum CYFRA21-1 with DC after the second cycle of chemotherapy.^[26,27]^ However, data are lacking on early prediction of DC after the first cycle of chemotherapy because the radiographic evaluation did not follow the current RECIST criteria, but used the older World Health Organization (WHO) standard.^[25,27]^ Additionally, the inclusion criteria did not require an elevated serum CYFRA21-1 level.^[25–27]^ In this study, we retrospectively analyzed the predictive value of percentage change of serum CYFRA21-1 before and after the first cycle of chemotherapy for radiologic DC according to the RECIST criteria. Ninety-seven patients with advanced NSCLC and elevated serum CYFRA21-1 were included. The aim of early prediction of chemotherapeutic effectiveness is to help identify patients who would benefit from a change in treatment.

## 2 Methods

### 2.1 Patient characteristics

**Figure 1:**
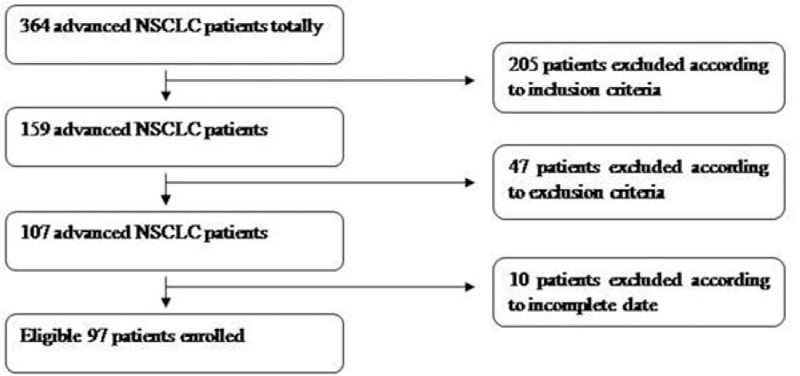
Flowchart of study participant selection.

A group of 97 patients treated for advanced NSCLC at Zhejiang Provincial People's Hospital between January 2009 and September 2014 were retrospectively analyzed. The selection procedure is shown in Fig. 1.The local ethics committee approved the study protocol. Eligible patients had histologically or cytologically confirmed stage IIIb or stage IV cancer that was newly diagnosed, or recurrent NSCLC that had not yet been treated. Other inclusion criteria were receipt of ≥2 cycles of chemotherapy with 2 platinum-containing drugs as first-line therapy. The choice of chemotherapy was at the discretion of medical oncologists. The disease was evaluated using RECIST version 1.1 criteria, ^[6]^ tumor response was assessed by imaging, and all patients had elevated serum CYFRA21-1 (≥3.8 μg/L) before or after the first cycle of chemotherapy. Cases without loss of follow-up data were evaluated. Patients with symptomatic brain metastasis, stage IIIb disease receiving concurrent chemoradiation were excluded.

### 2.2 CYFRA21-1 assay and calculation of percentage change

Serum samples (3 mL) were collected from the NSCLC patients in the week before the first cycle of chemotherapy (pre-CYFRA21-1) and the week before the second cycle of chemotherapy (post-CYFRA21-1). CYFRA21-1 was assayed by electrochemiluminescence (Roche E170 Immunology Analyzer) and calibrated using a commercially available CYFRA21-1 antigen (Bio-Rad Laboratories). The cut-off value of the normal serum CYFRA21-1 level was 3.8 μg/L based on the 95% confidence interval (CI) of the general Chinese population. The percentage change of serum CYFRA21-1 concentration was calculated as ([post-CYFRA21-1 − pre-CYFRA21-1]/pre-CYFRA21-1) × 100.

### 2.3 Assessment of chemotherapeutic effectiveness

The assessment of chemotherapeutic effectiveness was according to RECIST version 1.1 criteria ^[6]^ after the second cycle of chemotherapy. Patients who achieved CR, PR, and SD comprised a DC group, and the remaining patients comprised a PD group. Chemotherapeutic effectiveness was evaluated radiologically by computed tomography (CT) scans of the chest and superior abdomen conducted before the first and the third cycle of chemotherapy.

### 2.4 Statistical analysis

Categorical data were expressed as the number and percentage of cases, and chi-square or Fisher exact test was used to compare patients with DC and those with PD. The normality of continuous data distributions was assessed by the Kolmogorov–Smirnov test before statistical analysis. If the data had a normal or near-normal distribution, they were expressed as means ± standard deviation (SD), and independent sample *t* tests were used to compare the differences observed in the subjects with DC and PD. Data that did not have a normal distribution were expressed as medians and first (Q_1_) and third (Q_3_) quartiles; the Mann–Whitney *U* test was used to compare differences between the 2 groups.

To determine the association of serum CYFRA21-1 concentration and effectiveness of chemotherapy, the 97 patients were stratified into 3 groups by the percentage change in serum CYFRA21-1. With adjustments for age, sex, Eastern Cooperative Oncology Group (ECOG) performance status (PS) score, weight loss, cigarette smoking, histological type of cancer, gross type, clinical stage, and chemotherapy regimens, a multiple generalized linear model (GLM) and linear-trend test were performed to determine whether the percentage change in serum CYFRA21-1 was significantly associated with the effectiveness of treatment. Furthermore, receiver-operating characteristic (ROC) curves were plotted to assess the cut-off value of the percentage change in serum CYFRA21-1 in predicting effectiveness. The ability to accurately identify patients with better treatment response using the serum CYFRA21-1 value was determined by sensitivity and specificity estimated obtained by the area under the ROC curve (AUC) statistic. To determine the optimal cut-off value of the percentage change of serum CYFRA21-1 for screening the highly responsive NSCLC patients, we chose the point on the ROC curve that represented the highest sensitivity and specificity. We evaluated the potential optimal cut-off percentage change in serum CYFRA21-1 change by screening the highly responsive patients. All tests were 2-sided and *P* ≤ 0.05 was set as statistically significant. Data management and all statistical analyses were performed by using SAS version 9.4 software (SAS Institute Inc., Cary, NC).

## 3 Results

### 3.1 Patient characteristics and effectiveness of chemotherapy

The 97 NSCLC study participants included 15 with PD, 36 with PR, and 46 with SD. The patients had received 2 to 6 cycles of chemotherapy. Most patients received gemcitabine and cisplatin (GP) regimens or received gemcitabine and carboplatin (GC) regimens. The demographic and clinical characteristics of the patients and the effectiveness of NSCLC chemotherapy are shown in Table 1. The percentage change in serum CYFRA21-1 and the prevalence of patients with weight loss ≥5% were both significantly greater in those with PD than in those with DC. Between-group differences in other variables including age, sex, ECOG PS score, cigarette smoking, histological type, gross type, clinical stage, and chemotherapy regimens did not reach significance. Association between change of CYFRA21-1 and efficacy of chemotherapy was described in Supplemental Table 1.

**Table 1:**
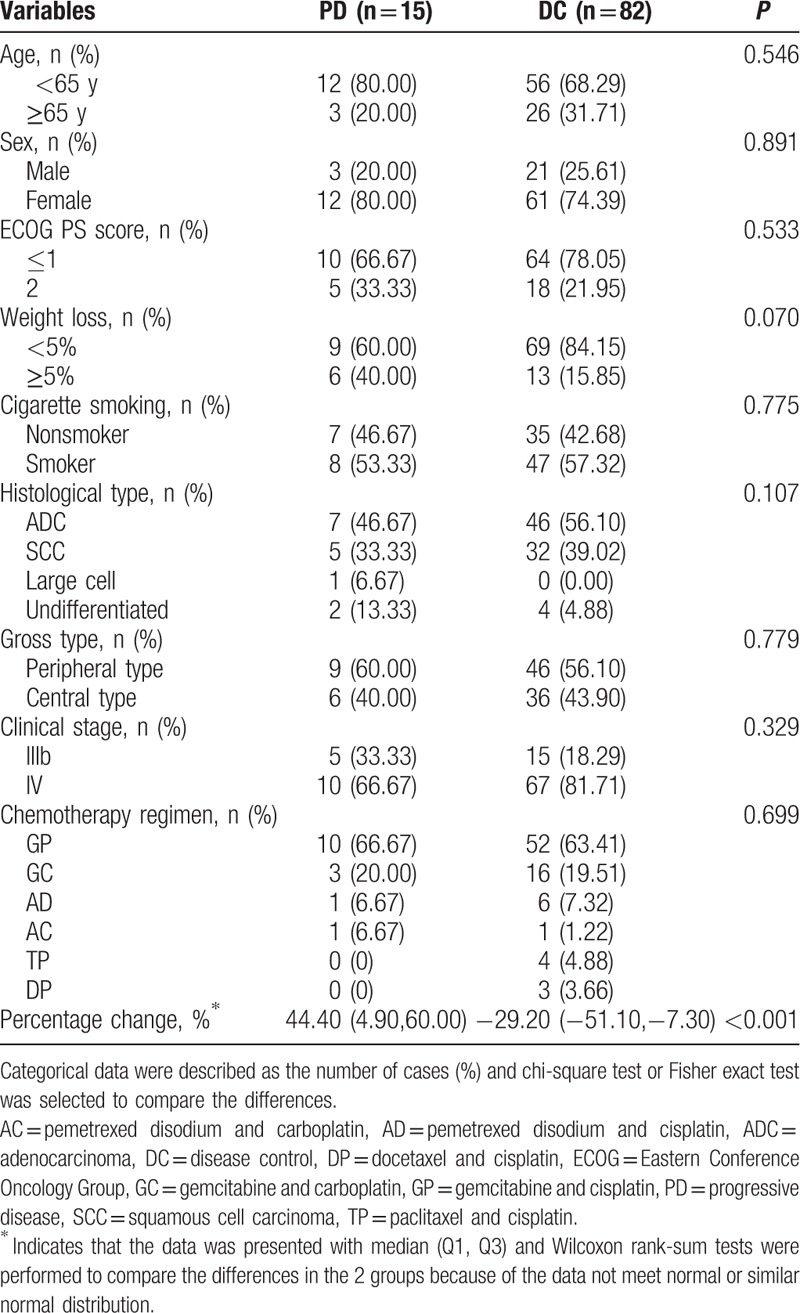
Characteristics of subjects by the efficacy of chemotherapy in NSCLC patients.

### 3.2 Association between serum CYFRA21-1 and chemotherapy effectiveness

**Figure 2:**
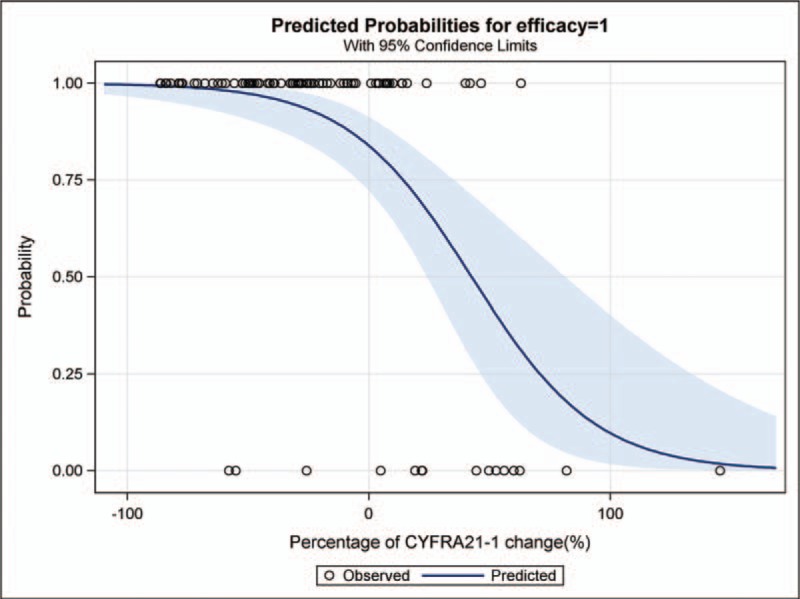
Predicted probability of chemotherapy effectiveness with the percentage change of serum CYFRA21-1.

The effect of serum CYFRA21-1 on prediction of the effectiveness of chemotherapy, as revealed by GLM, is shown in Table 2. The prevalence of DC in each of the 3 groups stratified by the percentage change of serum CYFRA21-1 was 93.80%, 97.00%, and 62.50% for decreases of 86.5%, 41.8%, and 6.4%, respectively. With adjustment for potential confounders including age, sex, ECOG PS score, weight loss, cigarette smoking, histological types of cancer, gross type, clinical stage, and chemotherapy regimens, the probability of DC was independently and negatively associated with the percentage change in serum CYFRA21-1. The lowest percentage change in serum CYFRA21-1 was associated with a significantly decreased probability of chemotherapy effectiveness (*P* = 0.012; Table 2). A negative monotonic relationship between the effectiveness of chemotherapy and the percentage change of CYFRA21-1 was also observed (*P*_trend_ = 0.002 in model 1 and *P*_trend_ = 0.02 in model 2; Table 2 and Fig. 2), confirming that the percentage change of serum CYFRA21-1 was an independent predictor, that is, a marker of probable chemotherapy effectiveness. Consistent findings on change of CYFRA21-1 were shown in Supplemental Table 2 and Supplemental Fig. 1.

**Table 2:**
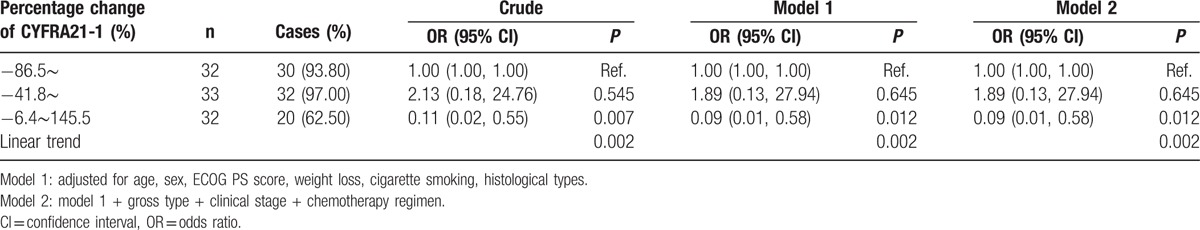
Individual effects of the percentage change of serum CYFRA21-1 (tertile) on prediction of the efficacy of chemotherapy for NSCLC.

### 3.3 Estimated optimal cut-off percentage change of serum CYFRA21-1 for prediction of chemotherapy effectiveness

**Figure 3:**
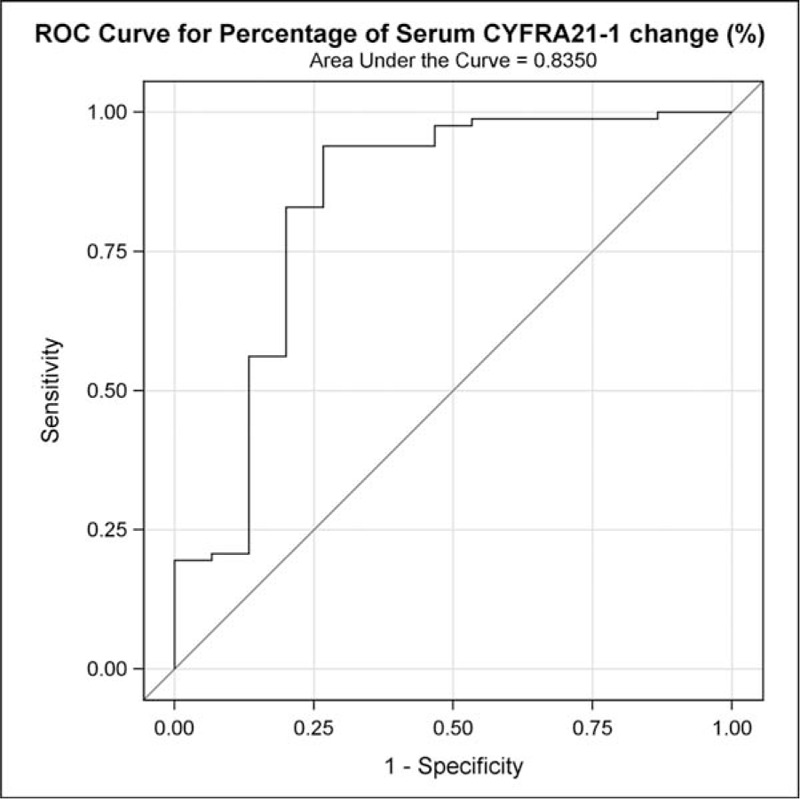
Receiver-operating characteristic (ROC) curve and area under the curve (AUC) showing the sensitivity and specificity of percentage change of serum CYFRA21-1 to predict chemotherapy effectiveness.

To estimate the optimal percentage change cut-off value for predicting probable DC, we chose the point, based on the ROC analysis, which had the greatest combined specificity and sensitivity. As shown in Fig. 3 and Table 3, the AUC statistic (95% CI) for the optimal percentage change was 0.84(0.69–0.98). After adjusting for potential confounding factors, the optimal cut-off point for predicting early DC after the first cycle of chemotherapy was a 17.5% increase in serum CYFRA21-1 (*P *< 0.001).

**Table 3:**
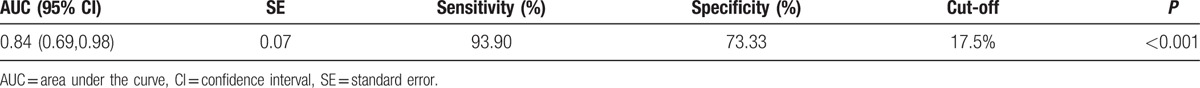
The prediction of chemotherapy efficacy by the percentage change of serum CYFRA21-1.

## 4 Discussion

Advances in treatment of malignant tumors have increased the need for tools for early evaluation of therapy effectiveness and optimization of patient management. In advanced NSCLC, CYFRA21-1 has value in predicting radiologic OR to chemotherapy after the first or second treatment cycle.^[25,28]^ In this study, we evaluated the relationship of percentage change in serum CYFRA21-1 after the first cycle of chemotherapy with radiologic DC in advanced NSCLC patients. Association between the change of CYFRA21-1 and the progression of NSCLC were also investigated and we found a good consistency as the percentage change of serum CYFRA21-1 (Supplemental Table 1, Supplemental Table 2, and Supplemental Fig. 1). As the value of CYFRA21-1 change would be significantly affected by its baseline level, so we do not think it was a good option like the percentage change of the serum CYFRA21-1, and the related results were not provided in the main text of the manuscript.

The results in Table 1 indicate after the first cycle of chemotherapy, the percentage change in serum CYFRA21-1 in the DC subgroup resulted in concentrations that were significantly lower than those observed in the PD subgroup (*P *< 0.001). Two recent studies by Holdenrieder et al reported similar changes in serum CYFRA21-1 concentration.^[29,30]^ The GLM models revealed that a negative percentage change in the serum CYFRA21-1 concentration was independently associated with the effectiveness of chemotherapy in these NSCLC patients (*P* = 0.012; Table 2). In addition, linear trend analysis also confirmed a negative monotonic relationship between the effectiveness of chemotherapy and the change in CYFRA21-1 concentration (Table 2 and Fig. 2). Thus, small percentage decreases (ie, a low negative percentage change) in concentration indicated a decreased probability of DC. The few previous studies of the association between the change in serum CYFRA21-1 and radiologic ORR after 2 cycles of chemotherapy have not been consistent. Yang et al^[23]^ found a positive association between radiologic OR and CYFRA 21-1 response (≥20% reduction over baseline level) by logistic analysis, but Hamzaoui et al^[24]^ reported that there was no correlation between change of CYFRA21-1 level and radiologic ORR in a series of 63 patients with advanced NSCLC. The lack of association between change in CYFRA21-1 and radiologic ORR might have been related to a cut-off value that was not determined by ROC curve analysis and the small number of patients. The negative monotonic relationship between the effectiveness of chemotherapy and the percentage change in CYFRA21-1 was observed in both model 1 (*P*_trend_ = 0.002) and model 2 (*P*_trend_ = 0.02; Table 2 and Fig. 2). These analyses confirm that the percentage change in serum CYFRA21-1 change was an independent predictor or potential marker of chemotherapy effectiveness.

The identification of the optimal percentage change cut-off in serum CYFRA21-1 is a key result, and it was found to be effective in predicting DC or PD (*P *< 0.001; Table 3 and Fig. 3) after the first cycle of chemotherapy. The 17.5% cut-off value had a high sensitivity (93.90%) and specificity (73.33%), and ROC curve analysis showed it efficiently distinguished advanced NSCLC patients with radiologic DC or PD early in clinical practice.

The optimum CYFRA21-1 cut-off value in this patient series was a 17.5% increase after the first cycle of chemotherapy, but a previous study reported that a 35% decline of CYFRA21-1 was the optimum cut-off value after the second cycle of chemotherapy.^[26]^ This study reached the same conclusion as the previous one, but there was a difference in the cut-off value. The reasons for this inconsistency may be related to the difference between DC and PD in the radiologic RECIST version 1.1 criteria, a 20% increase in long diameter that implies an increase of tumor burden associated with an increase of serum CYFRA21-1. This study applied the RECIST1.1 criteria, as they are now the gold standard, but the previous study followed WHO criteria. The RECIST PD criteria are more stringent,^[6]^ and may involve a greater increase of tumor burden when the evaluation is PD leading to an increase of cut-off value. Finally, the interval evaluated in this study was pretreatment to completion of the first cycle of chemotherapy and the interval in the previous study^[26]^ was to the end of the second cycle of chemotherapy, and the difference in timing might have influenced the cut-off value. In light of this, the cut-off value determined in this study is credible, practical, and predictive.

## 5 Conclusions

In conclusion, the percentage change of serum CYFRA21-1 after the first cycle of chemotherapy effectively predicted DC or PD in advanced NSCLC patients with elevated serum CYFRA21-1. The cut-off value of 17.5% may help clinicians to conveniently predict the treatment response, effectiveness of chemotherapy, or PD radiologically by CT earlier than in the past. An increase in serum CYFRA21-1 of 17.5% or more in advanced NSCLC patients receiving chemotherapy might permit adjusting the treatment regimen sooner than possible with traditional radiologic evaluation. In contrast, if the effectiveness of chemotherapy is supported by radiologic DC, then patients could continue with the original chemotherapy regimen.

Due to the limitations of retrospective studies and small cohorts of eligible patients, large, multicenter, randomized controlled clinical trials should be carried out to confirm the conclusion of this study and to determine a more precise cut-off value. The next steps are to study the relation of CYFRA21-1 change and prognosis, dynamic CYFRA21-1 change and chemotherapy effectiveness, and changes of additional serum tumor markers with chemotherapy outcome.

## Acknowledgment

We gratefully acknowledge Associate Professor Wenquan Niu of RuiJin Hospital, Shanghai Jiao Tong University School of Medicine, for his valuable suggestions in the manuscript preparation.

## Supplementary Material

Supplemental Digital Content
