# Supplementary material for: CYFRA 21-1 is an early predictor of chemotherapeutic effectiveness in advanced nonsmall cell lung cancer: An observational study
Source: Medicine (Baltimore). 2016 Dec 30;95(52):e5748. doi: 10.1097/MD.0000000000005748 (PMC5207583; doi:10.1097/MD.0000000000005748)
Supplement: Supplemental Digital Content [file medi-95-e5748-s001.docx]

Supplemental Figure 1. Predicted probability of chemotherapy effectiveness with the change of serum CYFRA21-1.


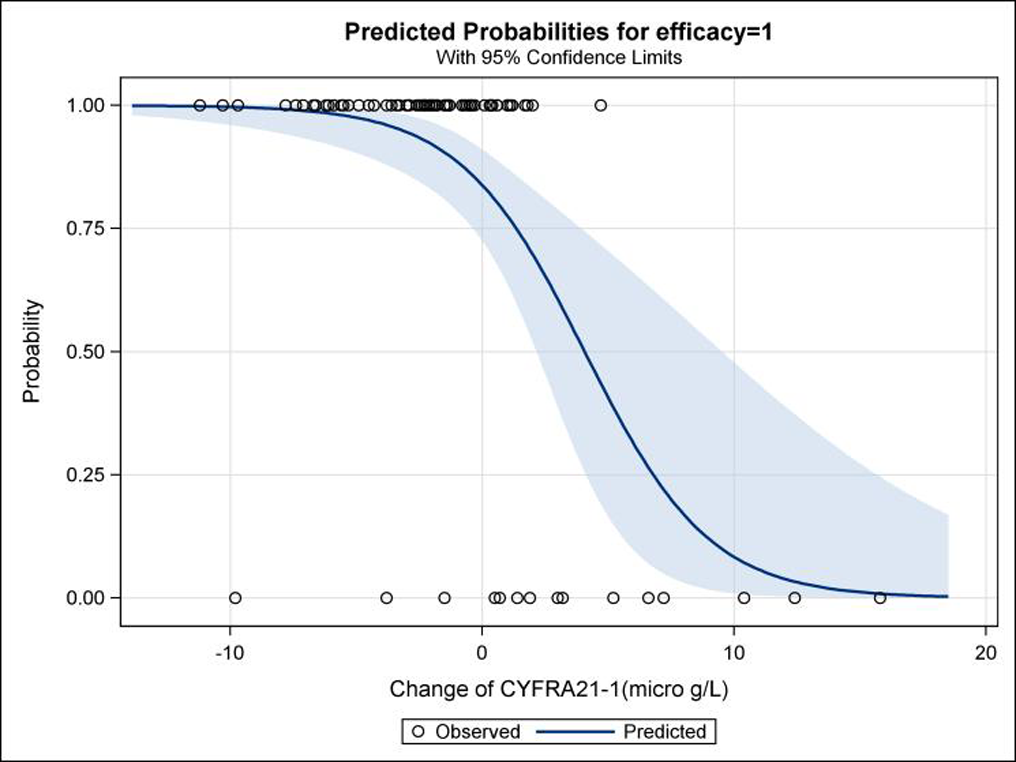


**Supplemental table 1. Association between change of serum CYFRA21-1 and the efficacy of chemotherapy in NSCLC patients**

| variables | DC  (n=82) | PD  (n=15) | P |
| --- | --- | --- | --- |
| Change of CYFRA21-1 ^ς^ | -2.00(-4.90,-0.40) | 3.00(0.50,7.20) | <0.001 |

^ς^ indicate that the data was presented with median (Q1, Q3) and Wilcoxon rank sum tests were performed to compare the differences in the two groups because of the data not meet normal or similar normal distribution.

**Supplemental table 2. Individual effects of change of serum CYFRA21-1 (tertile) on prediction of the efficacy of chemotherapy for NSCLC**

| Variable1 | N | Cases (%) | Crude | |  | Model 1 | |  | Model 2 | |
| --- | --- | --- | --- | --- | --- | --- | --- | --- | --- | --- |
|  |  |  | OR(95%CI) | P |  | OR(95%CI) | P |  | OR(95%CI) | P |
| Change of CYFRA21-1  (µg/L) |  |  |  |  |  |  |  |  |  |  |
| -125.7～ | 33 | 31(93.90) | 1.00(1.00,1.00) | Ref. |  | 1.00(1.00,1.00) | Ref. |  | 1.00(1.00,1.00) | Ref. |
| -2.6～ | 31 | 30(96.80) | 1.94(0.17,22.48) | 0.598 |  | 1.82(0.13,25.04) | 0.653 |  | 1.97(0.13,30.45) | 0.627 |
| -0.4～15.8 | 33 | 21(63.60) | 0.11(0.02,0.56) | 0.007 |  | 0.09(0.02,0.53) | 0.008 |  | 0.08(0.01,0.60) | 0.014 |
| Linear trend |  |  |  | 0.002 |  |  | 0.002 |  |  | 0.003 |

Model 1: adjusted for age, sex, ECOG PS score, weight loss, cigarette smoking, histological types.

Model 2: model 1+ gross type + clinical stage + chemotherapy regimen.
